# The genetic relationship between human and pet isolates: a core genome multilocus sequence analysis of multidrug-resistant bacteria

**DOI:** 10.1186/s13756-024-01457-7

**Published:** 2024-09-20

**Authors:** Antonia Genath, Carolin Hackmann, Luisa Denkel, Anna Weber, Friederike Maechler, Axel Kola, Stefan Schwarz, Petra Gastmeier, Rasmus Leistner

**Affiliations:** 1 Institute of Hygiene and Environmental Medicine, Charité University Medicine Berlin, Berlin, Germany; 2 Berlin School of Public Health, Charité University Medicine Berlin, Berlin, Germany; 3 Unit 36, Respiratory Infection, Department of Infectious Disease Epidemiology, Robert Koch Institute, Berlin, Germany; 4 Institute of Microbiology and Epizootics, Centre of Infection Medicine, School of Veterinary Medicine, Freie Universität Berlin, Berlin, Germany; 5 Veterinary Centre for Resistance Research (TZR), School of Veterinary Medicine, Freie Universität Berlin, Berlin, Germany; 6 Division Gastroenterology, Infectious Diseases and Rheumatology, Medical Department, Charité University Medicine Berlin, Berlin, Germany

**Keywords:** One Health, Multidrug-resistance, Molecular typing, cgMLST

## Abstract

**Introduction:**

The global increase of multidrug-resistant organisms (MDROs) is one of the most urgent public health threats affecting both humans and animals. The One Health concept emphasizes the interconnectedness of human, animal and environmental health and highlights the need for integrated approaches to combat antimicrobial resistance (AMR). Although the sharing of environments and antimicrobial agents between companion animals and humans poses a risk for MDRO transmission, companion animals have been studied to a lesser extent than livestock animals. This study therefore used core genome multilocus sequence typing (cgMLST) to investigate the genetic relationships and putative transmission of MDROs between humans and pets.

**Methods:**

This descriptive integrated typing study included 252 human isolates, 53 dog isolates and 10 cat isolates collected from 2019 to 2022 at the Charité University Hospital in Berlin, Germany. CgMLST was performed to characterize methicillin-resistant *Staphylococcus aureus*, vancomycin-resistant enterococci and multidrug-resistant gram-negative bacteria. The genetic diversity of the MDROs of the different host populations was determined and compared based on sequence type and core genome complex type.

**Results:**

Within this study the majority of samples from pets and humans was genetically distinct. However, for some isolates, the number of allelic differences identified by cgMLST was low. Two cases of putative household transmission or shared source of VR *E. faecium* and MDR *E. coli* between humans and pets were documented.

**Conclusions:**

The interaction between humans and their pets appears to play a minor role in the spread of the MDROs studied. However, further research is needed. This study emphasizes the importance of comprehensive molecular surveillance and a multidisciplinary One Health approach to understand and contain the spread of MDROs in human and animal populations.

**Trial Registration:**

The study is registered with the German Clinical Trials Register (DRKS00030009).

**Supplementary Information:**

The online version contains supplementary material available at 10.1186/s13756-024-01457-7.

## Introduction

Multidrug-resistant organisms (MDROs) pose a significant and urgent global public health threat to humans and animals. In 2019, the World Health Organization (WHO) ranked antimicrobial resistance (AMR) among the top ten threats to global health, with an estimated 4.95 million deaths worldwide attributed to AMR, 1.27 million of which are directly related to bacterial AMR [1, 2]. The consequences are ineffective treatments leading to prolonged illness, increased morbidity and mortality and increased healthcare costs [3–5]. However, there are also negative impacts on animal health, livestock production and food security [4, 6]. Combating AMR and its consequences therefore requires coordinated efforts in all areas of human, animal, and environmental health, in line with the One Health concept.

The One Health concept underlines the interdependence of human, animal and environmental health and emphasizes their mutual influence [7]. Although the importance of a One Health approach for AMR in livestock has been widely researched, it has received less attention in companion animals [8]. Nevertheless, companion animals and humans share a common living environment, and the same antimicrobial classes are used in both [9]. The close relationship and interactions between humans and pets enable the transmission of zoonotic, antimicrobial-resistant strains between both host populations, with both potentially serving as reservoirs for MDROs [10–12]. This emphasizes the importance of understanding the genetic diversity and relatedness of these pathogens and the need for integrated and molecular surveillance of antimicrobial-resistant bacterial populations in both human and animal populations to understand and mitigate the risk of transmission between these hosts.

Recent advances in whole genome sequencing (WGS) techniques have enabled comparative genomic analysis of bacterial populations in different hosts [13]. These techniques are replacing previous methods such as multilocus sequence typing (MLST), which is known for its limited discriminatory power and lower informative value in population genetic analyses [14, 15]. Core genome multilocus sequence typing (cgMLST), which analyzes thousands of target genes, offers greater discriminatory power and more precise strain typing [14]. This has led to increased integration of molecular and genomic typing in surveillance at the national and international levels, as well as in current research [16, 17]. However, the use of cgMLST for comparative analyses of MDROs between humans and companion animals has remained rare to date.

Therefore, cgMLST analysis has been used to characterize the bacterial pathogens of the AMR-Pet study (“Antimicrobial resistant pathogens transmitted via pets”), focusing on MDROs that impact human and animal health [12, 18]. Thus, this study provides a snapshot of the genetic diversity of MDROs in humans and companion animals in Berlin and surrounding areas in Germany. By comparing isolates from these groups, this study explored genetic relationships, similarities and differences to understand the potential colonization by similar MDROs among these hosts. These findings may help to better describe the role of human-pet interactions in the spread of these bacteria in the community.

## Methods

### Bacterial isolates

Multidrug-resistant (MDR) isolates were included in this study. MDR was defined based on in vitro antimicrobial susceptibility testing as being not susceptible to at least one agent in three or more antimicrobial categories [19]. Methicillin-resistant *Staphylococcus aureus* (MRSA), vancomycin-resistant enterococci (VRE) and multidrug-resistant gram-negative bacteria (MDR-GNB) from humans and pets collected as part of the case‒control study AMR-Pet at the Charité University Hospital in Berlin, Germany, from 2019 to 2022 were analyzed. In the AMR-Pet study, pet contact and other MDRO risk factors were compared between MDRO-positive and MDRO-negative patients. For this, patients completed questionnaires and provided nasal and rectal swabs as well as samples from the throat and stool of their pets (dogs and cats) after discharge from hospital. Only pet owners, who made up 21.7% of all participants, and their pets were relevant for our study. For the detailed recruitment process as well as bacterial species identification and antimicrobial susceptibility testing, see Hackmann et al. [18, 20]. The isolates were stored at -80 °C. To put the isolates into epidemiological and clinical contexts, the available epidemiological metadata from the AMR-Pet study were evaluated in parallel to strain typing. Particular attention was given to the host populations (human, dog, cat), the date of sampling and the place of residence.

### Molecular biological methods

Genomic DNA was extracted from an overnight culture of the included isolates on blood agar at 37 °C using a QiaCube Connect with UltraClean Microbial DNA Isolation Kit (both from Qiagen, Hilden, Germany) according to the manufacturer’s instructions. The concentration and purity were checked using a biophotometer. A total of 72 samples were processed at the Institute for Hygiene and Environmental Medicine, Charité Berlin. For WGS, sequencing libraries were prepared using the Nextera XT DNA library preparation kit, and short-read sequencing was performed using MiSeq 250 paired-end sequencing (both from Illumina Inc., San Diego, USA) according to the manufacturer’s instructions. A total of 210 samples was sequenced at the licensed and certified sequencing service provider Microsynth (Balgach, Switzerland). Tagmentation library preparation and 2*150 bp sequencing (both from Illumina Inc., San Diego, USA) were utilized. The read qualities were assessed with FastQC with the default settings [21].

### Bioinformatic analyses and visualization

For cgMLST analysis, the raw sequencing reads were subjected to quality trimming and de novo assembly using SeqSphere + software (Ridom GmbH, Münster, Germany, v9.0.2 with default settings). Sequence types (STs, https://pubmlst.org/) and core genome complex types (CTs, https://www.cgmlst.org) were assigned via SeqSphere + using published typing schemes, except for *Enterobacter cloacae* complex, for which an ad hoc scheme was established. The cgMLST clusters were determined by gene-by-gene comparisons with species-specific transmission cut-offs matched to the default settings of the software. The specific settings for pathogens were as follows: for VR *Enterococcus faecium* (average number of 3,118 genes), the typing scheme of de Been et al. (1423 core genes) was used, where isolates with 20 allelic differences served as clonal cluster cut-off [22]. For MDR *Escherichia coli* (average number of 4,661 genes), a typing scheme with 2513 core genes was used, and cluster analysis was performed with a cut-off of 10 allelic differences [23]. The cgMLST scheme developed by Rossen and Harmsen for *Klebsiella pneumoniae sensu lato* (average number of 5,297 genes) contains 2358 core genes, and cluster analysis was performed with cluster cut-off of 15 allelic differences [24]. The analysis of MRSA (average number of 2,796 genes) was performed using the typing scheme of Mellmann and Harmsen (1861 core genes), and cluster analysis was performed with a cluster distance threshold of 24 allelic differences as cut-off [25]. Additional information on the protein A gene of *S. aureus* (*spa*) was extracted. For the *E. cloacae* complex, an ad hoc cgMLST scheme was built in SeqSphere + using the reference strain *E. cloacae* complex sp. with the average number of 4,736 genes *(FDA-CDC-AR-0132*; core genome: 668 alleles). A detailed description of the process can be found in the appendix S1. The threshold for clonal clusters was set at 10 allele differences based on procedures in the literature [26]. The average number of genes in each species was obtained from NCBI: https://www.ncbi.nlm.nih.gov/nucleotide/.

Neighbor joining trees (NJTs) were initially generated in SeqSphere + and subsequently annotated and visualized with iTOL software (version 6.8.1). The occurrence of the various STs and CTs was shown using absolute and relative frequencies for each host population.

## Results

### Study population

**Fig. 1:**
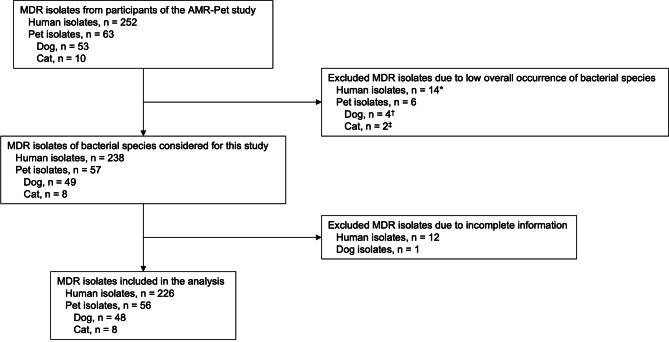
Flow chart of the isolates included in the study. List of excluded bacterial species: *Citrobacter spp.: 9, Enterobacter aerogenes: 1, Klebsiella oxytoca: 1, Proteus mirabilis: 1, Enterococcus faecalis: 3; †Citrobacter spp.: 2, Enterococcus cloacae: 1, Proteus penneri: 1; ‡Citrobacter spp.: 2

The study examined 252 MDRO isolates from humans, alongside 53 and 10 MDRO isolates from dogs and cats, respectively. These human isolates came from 626 pet owners who participated in the AMR-Pet study. Of these, 154 (24.6%) showed positive results for MDROs and were partially colonized with more than one bacterial species. Similarly, the pet isolates were obtained from 514 pets included in the study, with 62 (11.9%) testing positive for MDROs. In general, pets were colonized with only one MDRO, except for one dog that showed colonization by two different *E. coli* isolates. Of all the bacterial species detected among these isolates, five bacterial species that were present in humans and at least one pet species (dog or cat) were selected for comparison. These included MRSA, VR *E. faecium*, and selected species of MDR-GNB, more specifically, *E. coli*,* K. pneumoniae* and *E. cloacae* complex. Species that only occurred in one host population were excluded from further analysis to focus on the comparison between human and animal isolates. After excluding isolates with incomplete information, a total of 226 isolates from humans and 56 from pets (48 from dogs, eight from cats) were analyzed. A diagram illustrating the process of inclusion and exclusion of isolates is shown in Fig. 1.

Among these isolates, 16 (7.1%) human isolates and one (1.8%) pet isolate were MRSA. Furthermore, 105 (46.5%) of the human and five (8.9%) of the animal isolates were identified as VR *E. faecium*. MDR *E. coli* originated from 64 (28.3%) humans and 42 (75.0%) pets, MDR *K. pneumoniae* from 23 (10.2%) humans and one (1.8%) pet and MDR *E. cloacae* complex from 18 (8.0%) humans and seven (12.5%) pets. In 20 cases, both pet owners and their pets were MDRO-positive, but in only five of these cases humans and pets were colonized by isolates of the same species. Table 1 provides an overview of the distribution of the basic characteristics and MDROs among the humans and pets analyzed.

**Table 1:** Distribution of selected basic characteristics in the analyzed study population

Characteristic	Human *n* = 226	Pet *n* = 56
Sex (male)	*n* = 138 (61.1%)	*n* = 29 (51.8%)
Age*	58.9	7.7
Species (dog)	/	*n* = 48 (85.7%)
Species (cat)	/	*n* = 8 (14.3%)
Bacterial species		
*Enterococcus faecium* *Enterobacter cloacae* *Escherichia coli* *Klebsiella pneumoniae* *Staphylococcus aureus*	*n* = 105 (46.8%) *n* = 18 (7.7%) *n* = 64 (28.2%) *n* = 23 (10.5%) *n* = 16 (6.8%)	*n* = 5 (9.1%) *n* = 7 (12.4%) *n* = 42 (74.5%) *n* = 1 (1.8%) *n* = 1 (1.8%)
Owner-pet pairs		
*Counterpart missing* ^*†*^ *Counterpart MDRO-positive* *Counterpart MDRO-negative*	*n* = 129 (57.1%) *n* = 20 (8.8%) *n* = 77 (34.1%)	*n* = 0 (0%) *n* = 20 (35.7%) *n* = 36 (64.3%)

*Median (interquartile range (IQR)); ^†^Missing data occurred due to a low response rate from pet owners who failed to submit their pets’ samples after being sampled themselves during hospitalization despite two reminders

The microbiological analysis showed that most of the 105 human *Enterobacteriaceae* isolates were only resistant to 3rd generation cephalosporins (*n* = 45) or additionally to fluoroquinolones (*n* = 47), with three isolates being also resistant to carbapenems. Among the total of 50 *Enterobacteriaceae* pet isolates, 30 were only resistant to 3rd generation cephalosporins and 20 were also resistant to fluoroquinolones, with none being resistant to carbapenems. A high proportion of both groups (85.7% of human and 88.0% of animal isolates) produced an extended spectrum beta-lactamase (ESBL). The results are summarized in table S2 in the appendix.

### Strain typing: occurrence and comparative phylogenetic analysis

#### VR *E. faecium*

**Fig. 2:**
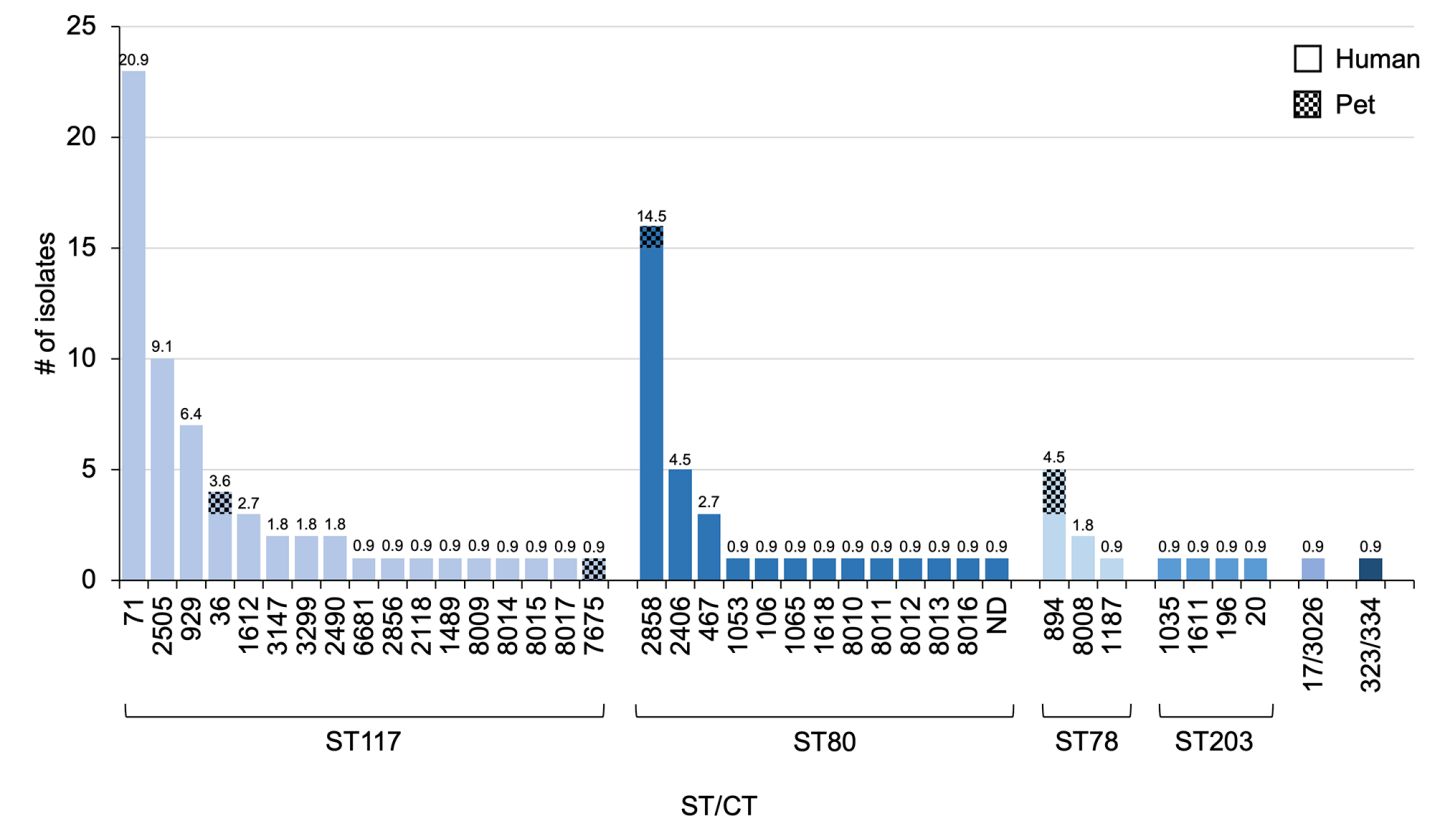
Absolute occurrence of sequence types (STs) and complex types (CTs) among all VR E. faecium isolates according to the cgMLST analysis. On the x-axis, the isolates are grouped according to their ST, and the individual bars correspond to the CTs. The number above the bars indicates the percentage of the respective CT among all VR E. faecium isolates. The patterned bar coloring indicates pet isolates. ND = CT could not be determined

A total of 110 VR *E. faecium* isolates from 105 humans, three dogs and two cats were analyzed by WGS. Only six STs were identified, with a relative dominance of ST117 (56.4%, *n* = 62) and ST80 (30.9%, *n* = 34). All STs (ST117, ST80, ST78, ST203, ST17 and ST323) were present in human isolates, three in dogs (ST117, ST80 and ST78) and two in cats (ST117 and ST78). A pet-specific ST did not occur. A total of 27 different CTs could be determined by cgMLST, 15 of which occurred only once. Within ST117, CT71 (20.9%, *n* = 23), CT2505 (9.1%, *n* = 10) and CT929 (6.4%, *n* = 7) formed the largest proportion and together accounted for more than one-third of all isolates. Among the second most frequent type, ST80, CT2858 (14.5%, *n* = 16) was by far the most common. Only the newly assigned ST117/CT7675 was pet-specific and occurred in one cat in our sample. A total of seven new CTs were assigned via cgmlst.org. For the complete distribution, see Fig. 2.

**Fig. 3:**
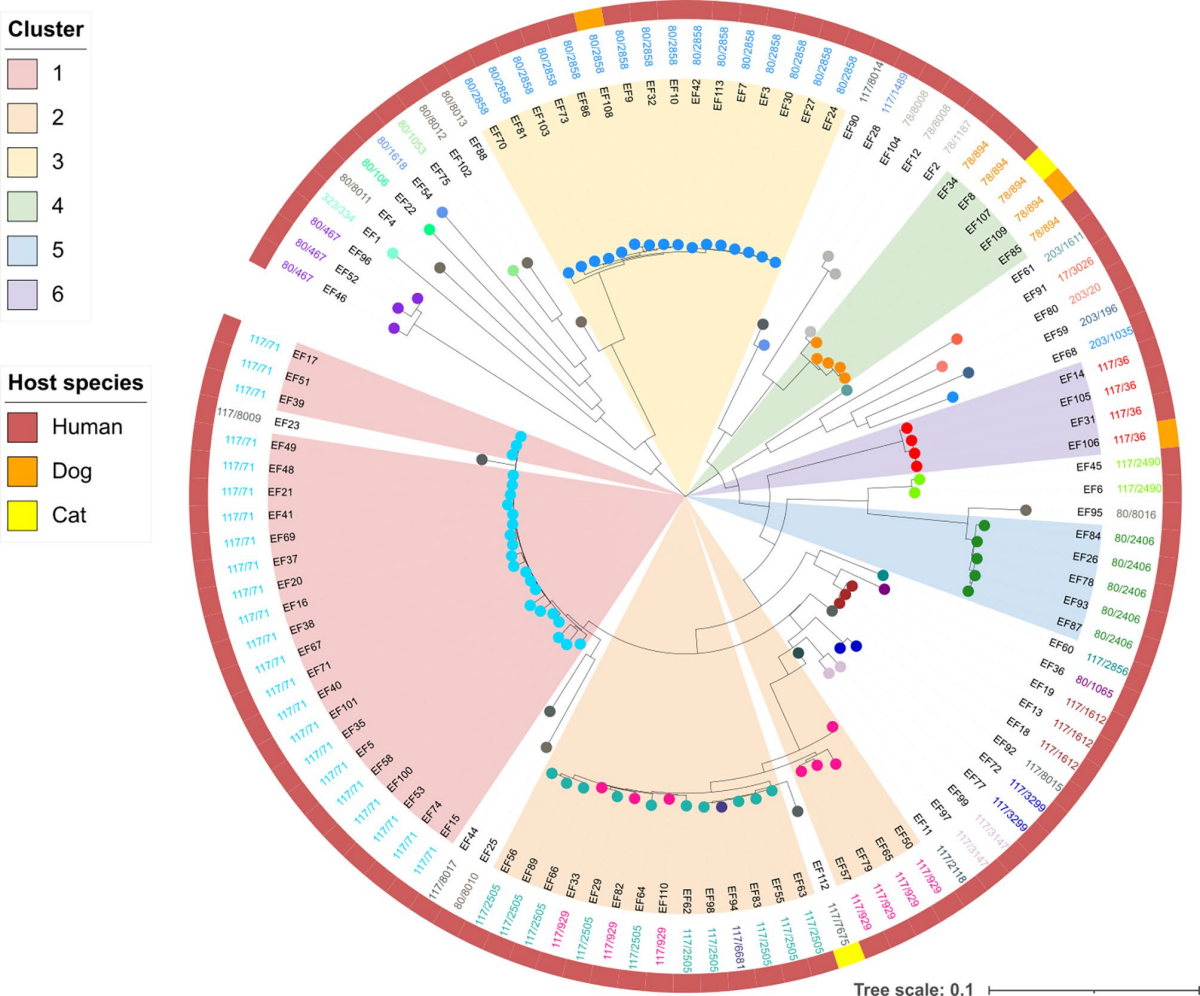
Neighbor joining tree based on 110 VR E. faecium core genomes of humans, dogs and cats. The phylogenetic tree and cluster analysis were initially performed using the software SeqSphere + and subsequently visualized and annotated in iTOL v6.8.1. The tree was rooted at the center. Combinations of sequence types (STs) and complex types (CTs) are indicated by colored circles at leaf nodes. The colors within the ranges indicate the different clusters. The outer ring represents the host populations (human, dog, cat). Cluster Threshold: ≤ 20 allelic differences

An NJT was created based on the comparison of task templates with SeqSphere + for the core genomes of isolates of humans, dogs and cats. The aim was to investigate the general overlap of STs or CTs between the host populations to determine possible transmission or shared sources. Except for one feline isolate, the pet isolates clustered with the human isolates with identical CTs with zero to four cgMLST allele differences to the nearest human isolate (Table S3 and Fig. 3).

Among all isolates, 12 different genetic clusters were observed, six of which contained more than three isolates. Cluster 1 (ST117/CT71) contained 23 and cluster 2 18 isolates (ten ST117/CT2505, seven ST117/CT929 and one ST117/CT6681), representing the largest clusters with only human isolates. An ST117/CT7675 isolate from a cat was not classified in cluster 3 due to a distance of only 23 alleles. Isolates of ST80/CT2858 were found in both humans and one dog. Phylogenetic analysis revealed a close relationship between the canine (EF108) and a human isolate (EF9) with a distance of only three alleles, although there was no known epidemiological link to be determined. Cluster 4 (ST78/CT894) comprised three human isolates and two pet isolates (one dog and one cat) with only three alleles distant from the nearest human isolate. In these cases, no epidemiological links between any of these isolates were known. ST117/CT36 (cluster 6) occurred in three isolates from humans and one from a dog, with no allelic differences detected between the isolates from two humans and the dog. The isolates EF105 and EF106 came from a owner-dog pair living in the same household, suggesting transmission. The remaining clusters contained exclusively human isolates.

#### MDR *E. coli*

**Fig. 4:**
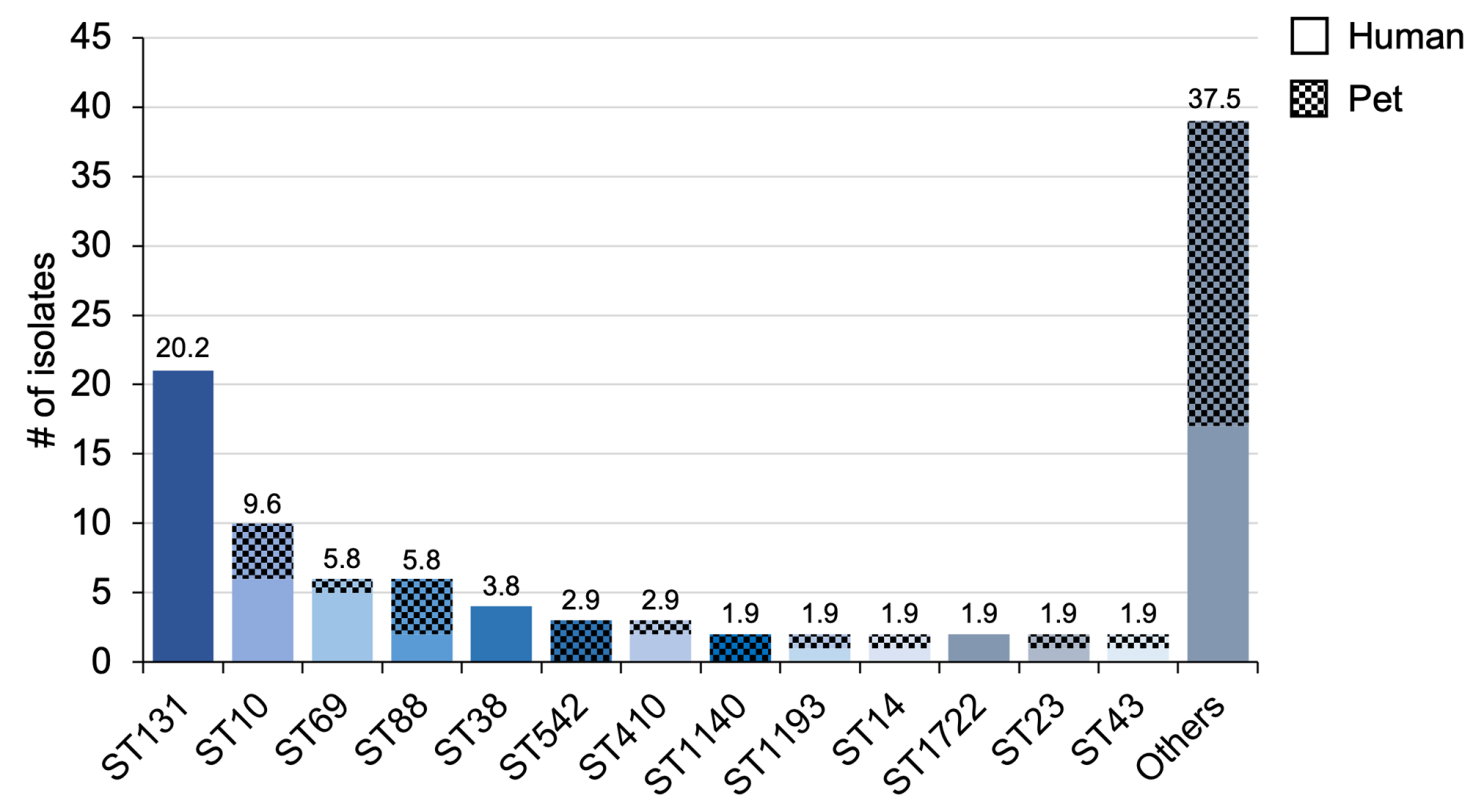
Absolute occurrence of sequence types (STs) among all MDR E. coli isolates according to MLST analysis. The number above the bars indicates the percentage of the respective ST among all isolates. The patterned bars indicate pet isolates. All STs that occurred only once are summarized as “Others”

The cgMLST analysis examined the genetic relationship between MDR *E. coli* isolates from 64 humans and 42 pets (38 dogs and four cats) in detail and revealed a very high clonal diversity: a total of 53 STs (Warwick scheme) were identified (Fig. 4). Most STs (84.9%) were associated with only one host population. The ST diversity of the isolates from companion animals was slightly greater than that of the human isolates (34 and 29 STs, respectively). Only 13 STs occurred at least twice. Among them, ST131 (20.6%, *n* = 22) was by far the most frequent and human-specific. The next most frequent types, ST10 (9.3%, *n* = 10), ST88 (6.5%, *n* = 7) and ST69 (5.6%, *n* = 6), occurred in all host populations. The most common pet-specific STs included ST542 (2.8%, *n* = 3) and ST1140 (1.9%, *n* = 2).

**Fig. 5:**
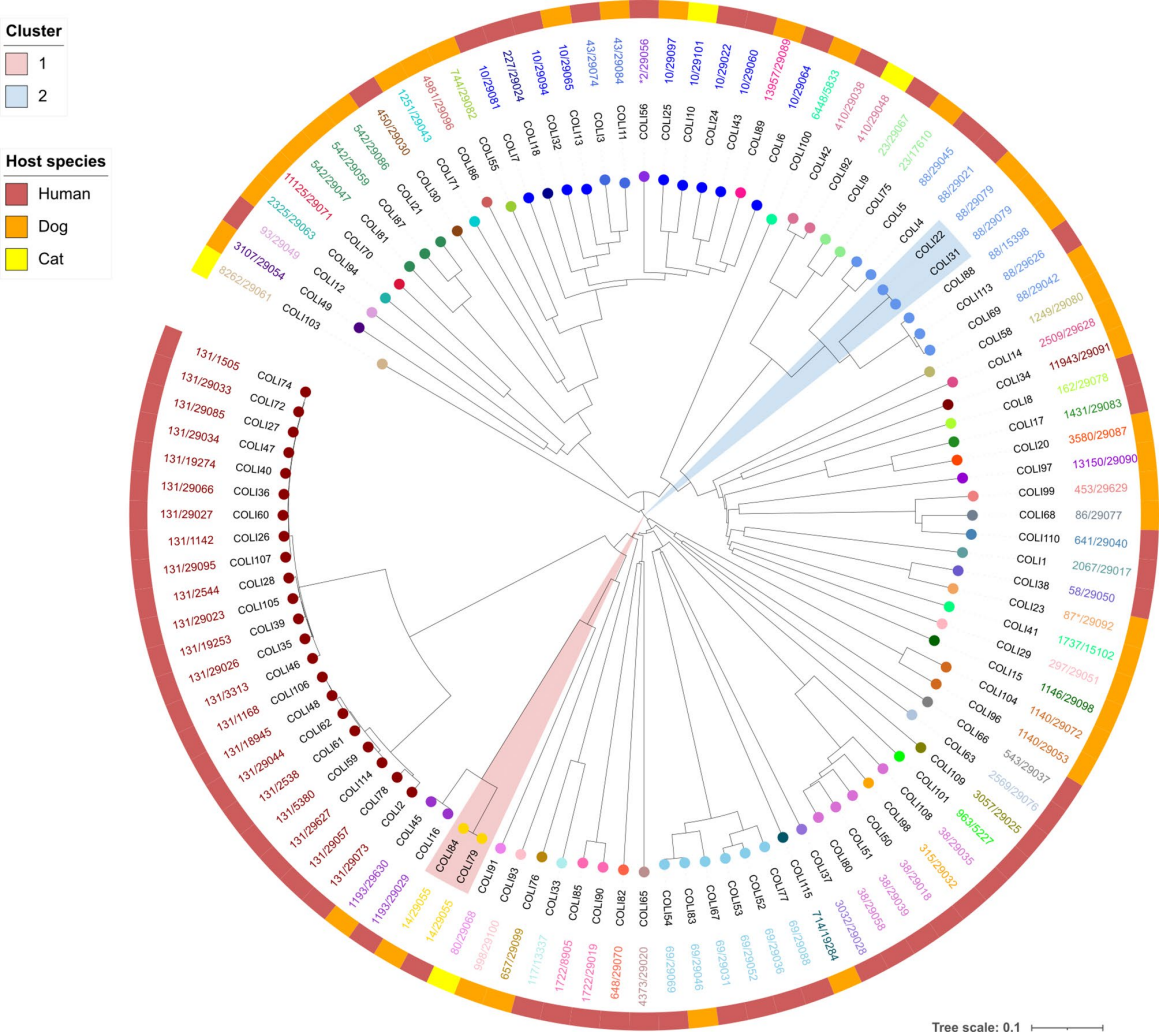
Neighbor joining tree based on MDR E. coli core genomes of 64 humans, 38 dogs and four cats. The phylogenetic tree and cluster analysis were created using SeqSphere + software and annotated in iTOL v6.8.1. The tree was rooted in the center. Combinations of sequence types (STs) and complex types (CTs) are indicated by colored circles at the leaf nodes. The colored ranges indicate the different clusters. The outer ring represents the host population (human, dog, cat). *No STs could be detected for the isolates with the study IDs COLI23 and COLI56 using the Warwick scheme. Therefore, the ST based on the Pasteur scheme is given here. Cluster Threshold: ≤ 10 allelic differences

Isolates of the same ST usually grouped together but showed variance, both in host-specific and multi-host STs (Fig. 5). For example, isolates within ST131 were divided into two groups, each with smaller allelic differences. In contrast, ST10 showed greater allelic differences between isolates and was interspersed with other STs from different host species. A total of 104 different CTs were identified in these 106 isolates, of which 90 (86.5%) were newly assigned via cgmlst.org. Most isolates were not clustered, only in two cases two isolates led to a cluster: ST14/CT29055 was detected in a pet owner and his dog from the same household, which may indicate transmission. ST88/CT29079 was isolated from two dogs without a known temporal or spatial connection. An overview of all CTs can be found in the appendix S4.

#### MDR *K. pneumoniae*

WGS was performed for MDR *K. pneumoniae* isolates from 23 humans and one dog. The typing revealed high clonal diversity, with a total of 14 STs that could be subdifferentiated into 18 CTs, with a relative dominance of ST1653/CT6052. Only ST1653, ST15 and ST307 occurred more than once, and together, they accounted for more than half of all the isolates (54.2%, *n* = 13). Several CTs were identified only within ST307, of which only CT606 occurred twice. In addition, seven new CTs were assigned. In the NJT, three genetic clusters were observed among all isolates, of which only cluster 1 (ST1653/CT6052) contained more than two isolates. In this cluster, exclusively human isolates with one to four allelic distances were grouped together. Temporal accumulation was observed here: all isolates were detected between the end of August and the beginning of November 2019. With one exception, the individuals were all from Berlin or the surrounding area. The remaining two clusters each contained two human isolates of ST15/CT1084 with four allele distances and ST307/606 with one allele distance. The only isolate from a dog (ST6544/CT10558) did not cluster with any of the human isolates and showed a distance of 1912 alleles to the closest human isolate. Details on the distribution of STs and CTs as well as the NJT can be found in figures S5 and S6 in the appendix.

#### MDR *E. cloacae* complex

**Fig. 6:**
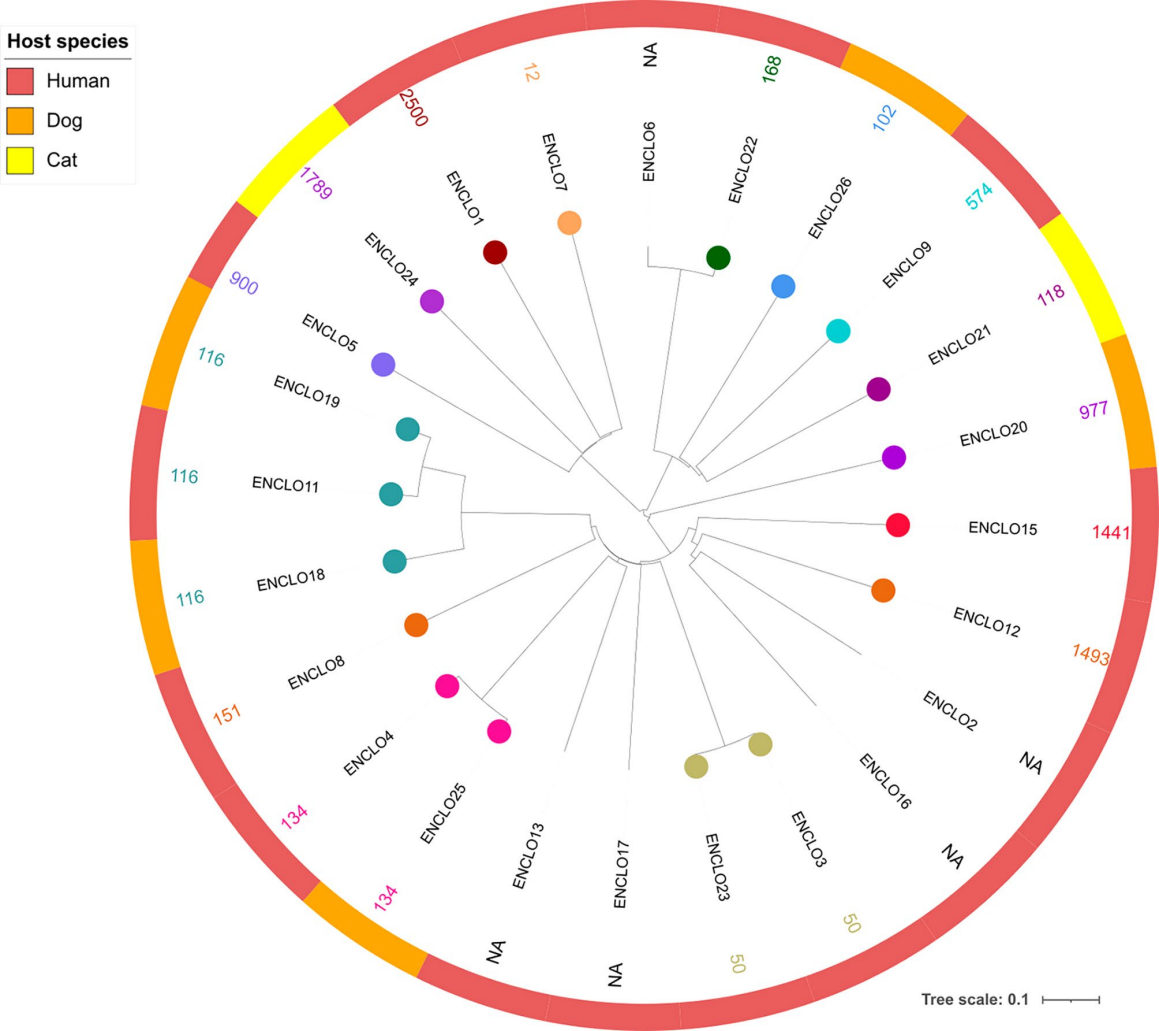
Neighbor joining tree of 25 MDR E. cloacae complex core genomes of 18 humans, five dogs and two cats. The phylogenetic tree and cluster analysis were created using SeqSphere + software and annotated in iTOL v6.8.1. The tree was rooted in the center. Sequence types (STs) – if applicable – are indicated by colored circles at the leaf nodes. The outer ring represents the host population (human, dog, cat). Cluster Threshold: ≤ 10 allelic differences

Twenty-five isolates (from 18 humans, five canines and two felines) were analyzed via WGS. A total of 20 isolates could be assigned to 16 STs, while no STs could be determined for five human isolates. Only ST116 (12.5%, *n* = 3), ST134 (8.3%, *n* = 2) and ST50 (8.3%, *n* = 2) occurred more than once. No CTs were defined by the ad hoc scheme; however, all three STs could be subdivided into several subgroups according to cgMLST. The analysis revealed a heterogeneous distribution of isolates differing between human and animal hosts, except for ST116 and ST134, where human and canine isolates grouped together (Fig. 6). Due to differences of consistently more than ten alleles within and between host populations, no clusters were formed. Relatively close relationships were identified between two human ST50 isolates with a difference of 29 alleles, a human and a canine ST134 isolate with an allelic distance of 35, and a human and a canine ST116 isolate with a difference of 60 alleles. Details on the distribution of STs can be found in figure S7 in the appendix.

#### MRSA

WGS was performed for MRSA isolates from 16 colonized humans and one dog. Five STs could be identified. These were divided into 16 CTs, 15 of which were new assignments via cgmlst.org. ST22 was dominant and accounted for almost two-thirds of all isolates (64.7%, *n* = 11). The cgMLST-based phylogeny revealed a distribution of isolates dominated by ST22, which was subdivided into 11 subgroups. Here, two epidemiologically unlinked human isolates (ST22/CT34786) formed the only cluster with a distance of 20 alleles. The only dog isolate (ST22/CT34789) was also grouped here with a distance of 72 alleles to the nearest human isolate. ST5 was represented by two isolates, which differed considerably by 323 alleles and were assigned to different CTs. Six different *spa* types occurred, with t032 being the dominant type (43.7%, *n* = 7). Details of the distribution of STs, CTs and *spa* types can be found in figure S8 in the appendix.

## Discussion

In this study, MDRO isolates from humans and pets were analyzed, using WGS and cgMLST to identify potential transmissions. Most MRSA, VRE and MDR-GNB showed high diversity, with minor overlaps between humans and pets. Only two human-pet pairs were colonized with strains of VR *E. faecium* and MDR *E. coli* that showed no allelic differences. This could indicate transmission or an acquisition from a common source, possibly within the household.

**VR *E. faecium*** was most prevalent in the human cohort of the study, which is consistent with the known high prevalence in Germany [27–30]. In contrast to the human isolates, VR *E. faecium* was less prevalent in our pets compared to other international samples [31].

The observed frequent occurrence of ST117/CT71 in humans is supported by the results of other studies [27–30]. In addition to the spread in and between hospitals, a contribution to its spread from other sources is suspected [27]. The results of this study, which showed no evidence of this CT in dogs and cats, at least do not support the hypothesis of a potential dissemination via pets. However, there were other genetic subtypes with overlaps between host populations, including ST117/CT36 with no allelic differences in a pet owner and their dog. Isolates from pets and humans also occurred together in other clusters (ST80/CT2858 and ST78/CT894). Although no epidemiological links between the isolates could be detected here, it cannot be excluded that certain CTs generally circulate between human and pet populations [32, 33].

The frequent occurrence of **MDR *E. coli*** in pets, especially dogs, corresponds to the high rates reported in the literature [34–36]. In humans, the prevalence slightly exceeded the levels of about 20% most recently reported by the German Antibiotic Resistance Surveillance (ARS) [37]. *E. coli* exhibits a great diversity of clonal lineages with dominant strains in humans and animals, reflecting the polyclonal distribution in our sample [35, 38, 39]. Thus, the dominant sequence types in our sample, ST131, ST10, ST88 and ST69, are currently frequently detected in nosocomial and community-associated infections [40–43]. The wide distribution of MDR *E. coli* in humans, animals and the environment suggests that their complex spread is due to different reservoirs and transmission routes [44]. In the literature, transmission routes between humans and pets are suspected and transmission events have been demonstrated [45–47]. However, most studies used typing methods with comparatively low discrimination, not sufficient to confirm clonal transfer [48, 49]. Using cgMLST, we observed different CTs within the same ST with greater allelic differences between humans and pets than within the respective host populations. Our observations underline the results by Pietsch et al. who hypothesized sporadic clonal transmission between host populations and subsequent independent adaptive microevolution [39, 44]. Overall, there have rarely been observed transmission events between humans and pets supporting the assumption that human-to-human transmission might be the predominant route of clonal spread in humans. [44, 45, 50]. However, humans and animals often share very similar resistance gene-carrying plasmids suggesting that this might be the predominant mode of resistance spread between humans and animals [39, 45, 49, 51].

**MDR *K. pneumoniae*** occurred more frequently in our human samples than reported in the literature, which may indicate an increasing public health burden at the participating study sites, whereas only one pet was colonized without evidence of transmission from humans [37, 52–55]. Our results confirm the high genetic diversity of MDR *K. pneumoniae*, with ST307 predominating. ST307 is known for its role in nosocomial outbreaks and significant plasmid conservation [53, 56–58]. The close genetic relationships among mostly human isolates indicate frequent transmission events possibly in the healthcare environment, which is suggested as main route of dissemination [59, 60]. Our results did not confirm previous findings in the literature regarding a possible transmission of MDR *K. pneumoniae* between humams and pets [57, 61].

**MDR *E. cloacae *complex** occurred slightly more frequent in our human sample than in previous studies [62–65]. In contrast, the proportion in pets in our study was significantly higher than the rates described in comparable studies [66, 67]. The isolates in our study had a diverse genetic background, which is consistent with findings in the literature [68–71]. This can be explained by sporadic transmission followed by rapid clonal divergence or common outbreaks [72]. However, the absence of temporal or geographic clustering suggests that common outbreaks were unlikely in our sample. The observed overlaps in STs indicate that clonal transmission between humans and animals is most likely of minor importance compared to the transmission of resistance genes [62].

The proportion of **MRSA** isolates in our sample was low in both humans and pets. This is in line with findings that the occurrence in humans in Germany has declined significantly during recent years and is generally low in pets [73–77]. Despite the diversity of clonal lineages, most human MRSA cases are dominated by few clonal lineages typically associated with healthcare settings types [76, 78–81]. The predominant clonal lineages in the human community also occur in dogs and cats, as confirmed by the ST22 isolate from our single dog sample [76, 80, 82–84]. However, it showed significant allelic differences to human isolates that may have resulted from the acquisition or loss of mobile genetic elements (MGE) and further host-specific mutations that allow spread into new host populations [83]. Transmission of MRSA between humans and pets has been proven in the past [76, 80, 85–88]. However, contact with healthcare facilities and human-to-human transmission are considered to be the main routes of colonization in humans, while pets are presumably affected by zoonotic infections of humans with an increased risk of MRSA [83, 85, 88].

### Limitations

This study has some limitations: it was limited to short-read sequencing. Thus, MGEs could not be investigated. A further limitation is that isolates from pets, particularly VR *E. faecium*, MDR *K. pneumoniae* and MRSA, were significantly less common than in humans. The lower number of MDROs from pet samples can be partly attributed to the generally lower prevalence in pets. In addition, almost half of the pet owners did not return the requested pet samples which reduced the sample size once more. The analysis of a larger sample could therefore yield different results. In the literature, highly discriminatory typing methods have only been used in recent years, limiting the comparability of results. Future studies should continue to use these methods in larger sample sizes to investigate possible overlaps and transmission pathways between the host populations.

## Conclusion

The data from this study provide a better understanding of the genomic epidemiology of MDROs in humans and pets in Germany. Most of the pet isolates showed a genetic background that differed from those of the human isolates. In addition, the overall MDRO prevalence in pets was low, especially with regard to clinically relevant human pathogens. Significantly more pet owners were tested MDRO-positive compared to pets. This suggests that the interaction between humans and their pets appears to play a minor role in the spread of the MDROs. Pets might act as spillover hosts rather than reservoirs for MDROs with transmission potential to humans. Yet, the possibility of transmission from a shared source remains. Future studies should include other possible transmission routes, common sources of resistant strains and horizontal transmission of plasmids.

## Electronic supplementary material

Below is the link to the electronic supplementary material.

Supplementary Material 1

Supplementary Material 2

Supplementary Material 3

Supplementary Material 4

Supplementary Material 5

Supplementary Material 6

Supplementary Material 7

Supplementary Material 8

Supplementary Material 9

Supplementary Material 10

Supplementary Material 11

Supplementary Material 12

Supplementary Material 13

Supplementary Material 14

## Data Availability

The sequence data have been submitted to the Sequence Read Archive (SRA) under submission number SUB14292542.

## Abbreviations

AMR: Antimicrobial resistance
AMR-Pet: Antimicrobial resistant pathogens transmitted via pets
ARS: German Antibiotic Resistance Surveillance
cgMLST: Core genome multilocus sequence typing
CT Core: Genome complex type
ESBL: Extended Spectrum Beta-Lactamase
IQR: Interquartile range
MDR: Multidrug-resistant
MDR-GNB: Multidrug-resistant gram-negative bacteria
MDRO: Multidrug-resistant organism
MGE: Mobile genetic elements
MLST: Multilocus sequence typing
MRSA: Methicillin-resistant Staphylococcus aureus
ND: Not determined
NJT: Neighbor joining tree
NRC: German National Reference Center
Spa: Staphylococcal protein A gene
ST: Sequence type
VR: Vancomycin-resistant
VRE: Vancomycin-resistant enterococci
WHO: World Health Organization
WGS: Whole genome sequencing
