# Supplementary material for: The genetic relationship between human and pet isolates: a core genome multilocus sequence analysis of multidrug-resistant bacteria
Source: Antimicrob Resist Infect Control. 2024 Sep 20;13:107. doi: 10.1186/s13756-024-01457-7 (PMC11416027; doi:10.1186/s13756-024-01457-7)
Supplement: Supplementary file 1 — Supplementary Material 1 [file 13756_2024_1457_MOESM1_ESM.docx]

Additional file 1

S 1: Description of the development of an ad hoc cgMLST scheme for E. cloacae complex

An ad hoc cgMLST scheme for *E. cloacae* complex was created in SeqSphere+ v9.0.2 using the default settings. *E. cloacae* complex sp. FDA-CDC-AR_0132 (NZ_CP027618.1) served as the seed genome. The query genomes were *E. cloacae* complex sp. strain FDAARGOS_77 (NZ_CP026975.1), *E. cloacae* complex sp. FDA-CDC-AR_0164 (NZ_CP028950.1), *E. cloacae* complex sp. strain N13-01531 (NZ_CP037919. 1), *E. cloacae* complex sp. ECL112 (NZ_CP077661.1 chromosome), *E. cloacae* complex sp. ECL72 (NZ_CP077659.1), and *E. cloacae* complex sp. ECL78 (NZ_CP077660.1).
