# Supplementary material for: The genetic relationship between human and pet isolates: a core genome multilocus sequence analysis of multidrug-resistant bacteria
Source: Antimicrob Resist Infect Control. 2024 Sep 20;13:107. doi: 10.1186/s13756-024-01457-7 (PMC11416027; doi:10.1186/s13756-024-01457-7)
Supplement: Supplementary file 2 — Supplementary Material 2 [file 13756_2024_1457_MOESM2_ESM.docx]

# Additional file 2

*S 2: Distribution of resistance among a) 226 human and b) 56 animal MDR isolates. ESBL = extended spectrum beta-lactamase.*

|  | **Total** | **Methicillin resistance** | **Vancomycin resistance** | **3^rd^ Generation Cephalosporine resistance** | **Fluorchinolone resistance** | **Carbapenem resistance** | **ESBL positive** |  |  |
| --- | --- | --- | --- | --- | --- | --- | --- | --- | --- |
| *Enterococcus faecium* | 105 | - | 105 | - | - | - | - |  |  |
| *Escherichia coli* | 64 | - | - | 31 | 33 | 0 | 61 |  |  |
| *Klebsiella pneumoniae* | 23 | - | - | 8 | 13 | 2 | 20 |  |  |
| *Enterobacter cloacae* complex | | 18 | - | - | 16 | 1 | 1 | 9 |  |
| *Staphylococcus aureus* | | | 16 | 16 | - | - | - | - | - |
| **Sum** | **226** | **16** | **105** | **45** | **47** | **3** | **90** |  |  |

a)

b)

|  | **Total** | **Methicillin resistance** | **Vancomycin resistance** | **3^rd^ Generation Cephalosporine resistance** | **Fluorchinolone resistance** | **Carbapenem resistance** | **ESBL positive** |  |  |
| --- | --- | --- | --- | --- | --- | --- | --- | --- | --- |
| *Enterococcus faecium* | 5 | - | 5 | - | - | - | - |  |  |
| *Escherichia coli* | 42 | - | - | 23 | 19 | 0 | 41 |  |  |
| *Klebsiella pneumoniae* | 1 | - | - | 0 | 1 | 0 | 1 |  |  |
| *Enterobacter cloacae* complex | | 7 | - | - | 7 | 0 | 0 | 2 |  |
| *Staphylococcus aureus* | | | 1 | 1 | - | - | - | - | - |
| **Sum** | **56** | **1** | **5** | **30** | **20** | **0** | **44** |  |  |
