# Supplementary material for: The genetic relationship between human and pet isolates: a core genome multilocus sequence analysis of multidrug-resistant bacteria
Source: Antimicrob Resist Infect Control. 2024 Sep 20;13:107. doi: 10.1186/s13756-024-01457-7 (PMC11416027; doi:10.1186/s13756-024-01457-7)
Supplement: Supplementary file 3 — Supplementary Material 3 [file 13756_2024_1457_MOESM3_ESM.docx]

Additional file 3

S 3: Results obtained from genomic typing of pet isolates (dogs, cats). The cgMLST allelic distance to closest human isolate was determined using the SeqSphere+ software.

| StudyID | MDRO | Host  species | Sequence  type | Complex  type | cgMLST allelic distance to closest human isolate |
| --- | --- | --- | --- | --- | --- |
| EF106 | *E. faecium* | Dog | 117 | 36 | 0 |
| EF108 | *E. faecium* | Dog | 80 | 2858 | 3 |
| EF107 | *E. faecium* | Cat | 78 | 894 | 3 |
| EF109 | *E. faecium* | Dog | 78 | 894 | 4 |
| EF112 | *E. faecium* | Cat | 117 | 7675 | 23 |
| COLI84 | *E. coli* | Dog | 14 | 29055 | 3 |
| COLI45 | *E. coli* | Dog | 1193 | 29630 | 37 |
| COLI83 | *E. coli* | Dog | 69 | 29046 | 37 |
| COLI69 | *E. coli* | Dog | 88 | 29042 | 49 |
| COLI88 | *E. coli* | Dog | 88 | 15398 | 69 |
| COLI92 | *E. coli* | Cat | 410 | 29048 | 89 |
| COLI75 | *E. coli* | Dog | 23 | 17610 | 222 |
| COLI23 | *E. coli* | Dog | 87* | 29092 | 326 |
| COLI68 | *E. coli* | Dog | 86 | 29077 | 344 |
| COLI11 | *E. coli* | Dog | 43 | 29084 | 354 |
| COLI20 | *E. coli* | Dog | 3580 | 29087 | 453 |
| COLI13 | *E. coli* | Dog | 10 | 29065 | 461 |
| COLI89 | *E. coli* | Dog | 13957 | 29089 | 480 |
| COLI22 | *E. coli* | Dog | 88 | 29079 | 487 |
| COLI31 | *E. coli* | Dog | 88 | 29079 | 487 |
| COLI10 | *E. coli* | Cat | 10 | 29101 | 519 |
| COLI55 | *E. coli* | Dog | 744 | 29082 | 588 |
| COLI25 | *E. coli* | Dog | 10 | 29097 | 657 |
| COLI76 | *E. coli* | Dog | 657 | 29099 | 711 |
| COLI99 | *E. coli* | Dog | 453 | 29629 | 716 |
| COLI86 | *E. coli* | Dog | 4981 | 29096 | 836 |
| COLI71 | *E. coli* | Dog | 1251 | 29043 | 1270 |
| COLI87 | *E. coli* | Dog | 542 | 29059 | 1406 |
| COLI49 | *E. coli* | Dog | 3107 | 29054 | 1547 |
| COLI97 | *E. coli* | Dog | 13150 | 29090 | 1556 |
| COLI94 | *E. coli* | Dog | 2325 | 29063 | 1578 |
| COLI15 | *E. coli* | Dog | 1146 | 29098 | 1605 |
| COLI91 | *E. coli* | Cat | 80 | 29068 | 1648 |
| COLI21 | *E. coli* | Dog | 542 | 29086 | 1658 |
| COLI58 | *E. coli* | Dog | 1249 | 29080 | 1666 |
| COLI29 | *E. coli* | Dog | 2787 | 29093 | 1865 |
| COLI100 | *E. coli* | Dog | 6448 | 5833 | 1939 |
| COLI93 | *E. coli* | Dog | 998 | 29100 | 2017 |
| COLI104 | *E. coli* | Dog | 1140 | 29072 | 2075 |
| COLI81 | *E. coli* | Dog | 542 | 29047 | 2092 |
| COLI103 | *E. coli* | Cat | 8262 | 29061 | 2125 |
| COLI115 | *E. coli* | Dog | 714 | 19284 | 2164 |
| COLI96 | *E. coli* | Dog | 1140 | 29053 | 2379 |
| COLI70 | *E. coli* | Dog | 11125 | 29071 | 2653 |
| COLI41 | *E. coli* | Dog | 1737 | 15102 | 3048 |
| COLI34 | *E. coli* | Dog | 11943 | 29091 | 3236 |
| COLI14 | *E. coli* | Dog | 2509 | 29628 | 4844 |
| KLPN1 | *K. pneumoniae* | Dog | 6544 | 10558 | 1912 |
| ENCLO25 | *E. cloacae* complex | Dog | 134 | / | 35 |
| ENCLO19 | *E. cloacae* complex | Dog | 116 | / | 60 |
| ENCLO18 | *E. cloacae* complex | Dog | 116 | / | 206 |
| ENCLO21 | *E. cloacae* complex | Cat | 118 | / | 440 |
| ENCLO24 | *E. cloacae* complex | Cat | 1789 | / | 507 |
| ENCLO20 | *E. cloacae* complex | Dog | 977 | / | 572 |
| ENCLO26 | *E. cloacae* complex | Dog | 102 | / | 888 |
| STAU9 | *S. aureus* | Dog | 22 | 34789 | 72 |
