# Supplementary material for: The genetic relationship between human and pet isolates: a core genome multilocus sequence analysis of multidrug-resistant bacteria
Source: Antimicrob Resist Infect Control. 2024 Sep 20;13:107. doi: 10.1186/s13756-024-01457-7 (PMC11416027; doi:10.1186/s13756-024-01457-7)
Supplement: Supplementary file 4 — Supplementary Material 4 [file 13756_2024_1457_MOESM4_ESM.docx]

Additional file 4

S 4: Core genome complex types of all MDR E. coli isolates from humans, dogs and cats. *No ST could be determined for the isolates with the study ID COLI23 and COLI56 using the Warwick scheme. Therefore, the ST based on the Pasteur scheme was given here.

| StudyID | Host species | ST Warwick | CT |
| --- | --- | --- | --- |
| COLI1 | Human | 2067 | 29017 |
| COLI2 | Human | 131 | 29073 |
| COLI3 | Human | 43 | 29074 |
| COLI4 | Human | 88 | 29021 |
| COLI5 | Human | 88 | 29045 |
| COLI6 | Human | 10 | 29064 |
| COLI7 | Human | 10 | 29081 |
| COLI8 | human | 162 | 29078 |
| COLI9 | Human | 23 | 29067 |
| COLI10 | Cat | 10 | 29101 |
| COLI11 | Dog | 43 | 29084 |
| COLI12 | Human | 93 | 29049 |
| COLI13 | Dog | 10 | 29065 |
| COLI14 | Dog | 2509 | 29628 |
| COLI15 | Dog | 1146 | 29098 |
| COLI16 | Human | 1193 | 29029 |
| COLI17 | Human | 1431 | 29083 |
| COLI18 | Human | 227 | 29024 |
| COLI20 | Dog | 3580 | 29087 |
| COLI21 | Dog | 542 | 29086 |
| COLI22 | Dog | 88 | 29079 |
| COLI23 | Dog | *87 | 29092 |
| COLI24 | Human | 10 | 29022 |
| COLI25 | Dog | 10 | 29097 |
| COLI26 | Human | 131 | 1142 |
| COLI27 | Human | 131 | 29085 |
| COLI28 | Human | 131 | 2544 |
| COLI29 | Dog | 2787 | 29093 |
| COLI30 | Human | 450 | 29030 |
| COLI31 | Dog | 88 | 29079 |
| COLI32 | Human | 10 | 29094 |
| COLI33 | Human | 117 | 13337 |
| COLI34 | Dog | 11943 | 29091 |
| COLI35 | Human | 131 | 29026 |
| COLI36 | Human | 131 | 29066 |
| COLI37 | Human | 3032 | 29028 |
| COLI38 | Human | 58 | 29050 |
| COLI39 | Human | 131 | 19253 |
| COLI40 | Human | 131 | 19274 |
| COLI41 | Dog | 1737 | 15102 |
| COLI42 | Human | 410 | 29038 |
| COLI43 | Human | 10 | 29060 |
| COLI45 | Dog | 1193 | 29630 |
| COLI46 | Human | 131 | 3313 |
| COLI47 | Human | 131 | 29034 |
| COLI48 | Human | 131 | 18945 |
| COLI49 | Dog | 3107 | 29054 |
| COLI50 | Human | 38 | 29018 |
| COLI51 | Human | 38 | 29039 |
| COLI52 | Human | 69 | 29036 |
| COLI53 | Human | 69 | 29052 |
| COLI54 | Human | 69 | 29069 |
| COLI55 | Dog | 744 | 29082 |
| COLI56 | Human | *2 | 29056 |
| COLI58 | Dog | 1249 | 29080 |
| COLI59 | Human | 131 | 5380 |
| COLI60 | Human | 131 | 29027 |
| COLI61 | Human | 131 | 2538 |
| COLI62 | Human | 131 | 29044 |
| COLI63 | Human | 2569 | 29076 |
| COLI65 | Human | 4373 | 29020 |
| COLI66 | Human | 543 | 29037 |
| COLI67 | Human | 69 | 29031 |
| COLI68 | Dog | 86 | 29077 |
| COLI69 | Dog | 88 | 29042 |
| COLI70 | Dog | 11125 | 29071 |
| COLI71 | Dog | 1251 | 29043 |
| COLI72 | Human | 131 | 29033 |
| COLI74 | Human | 131 | 1505 |
| COLI75 | Dog | 23 | 17610 |
| COLI76 | Dog | 657 | 29099 |
| COLI77 | Human | 69 | 29088 |
| COLI78 | Human | 131 | 29057 |
| COLI79 | Human | 14 | 29055 |
| COLI80 | Human | 38 | 29058 |
| COLI81 | Dog | 542 | 29047 |
| COLI82 | Human | 648 | 29070 |
| COLI83 | Dog | 69 | 29046 |
| COLI84 | Dog | 14 | 29055 |
| COLI85 | Human | 1722 | 8905 |
| COLI86 | Dog | 4981 | 29096 |
| COLI87 | Dog | 542 | 29059 |
| COLI88 | Dog | 88 | 15398 |
| COLI89 | Dog | 13957 | 29089 |
| COLI90 | Human | 1722 | 29019 |
| COLI91 | Cat | 80 | 29068 |
| COLI92 | Cat | 410 | 29048 |
| COLI93 | Dog | 998 | 29100 |
| COLI94 | Dog | 2325 | 29063 |
| COLI96 | Dog | 1140 | 29053 |
| COLI97 | Dog | 13150 | 29090 |
| COLI98 | Human | 315 | 29032 |
| COLI99 | Dog | 453 | 29629 |
| COLI100 | Dog | 6448 | 5833 |
| COLI101 | Human | 963 | 5227 |
| COLI103 | Cat | 8262 | 29061 |
| COLI104 | Dog | 1140 | 29072 |
| COLI105 | Human | 131 | 29023 |
| COLI106 | Human | 131 | 1168 |
| COLI107 | Human | 131 | 29095 |
| COLI108 | Human | 38 | 29035 |
| COLI109 | Human | 3057 | 29025 |
| COLI110 | Human | 641 | 29040 |
| COLI113 | Human | 88 | 29626 |
| COLI114 | Human | 131 | 29627 |
| COLI115 | Dog | 714 | 19284 |
