# Supplementary figures and images for: The genetic relationship between human and pet isolates: a core genome multilocus sequence analysis of multidrug-resistant bacteria
Source: Antimicrob Resist Infect Control. 2024 Sep 20;13:107. doi: 10.1186/s13756-024-01457-7 (PMC11416027; doi:10.1186/s13756-024-01457-7)

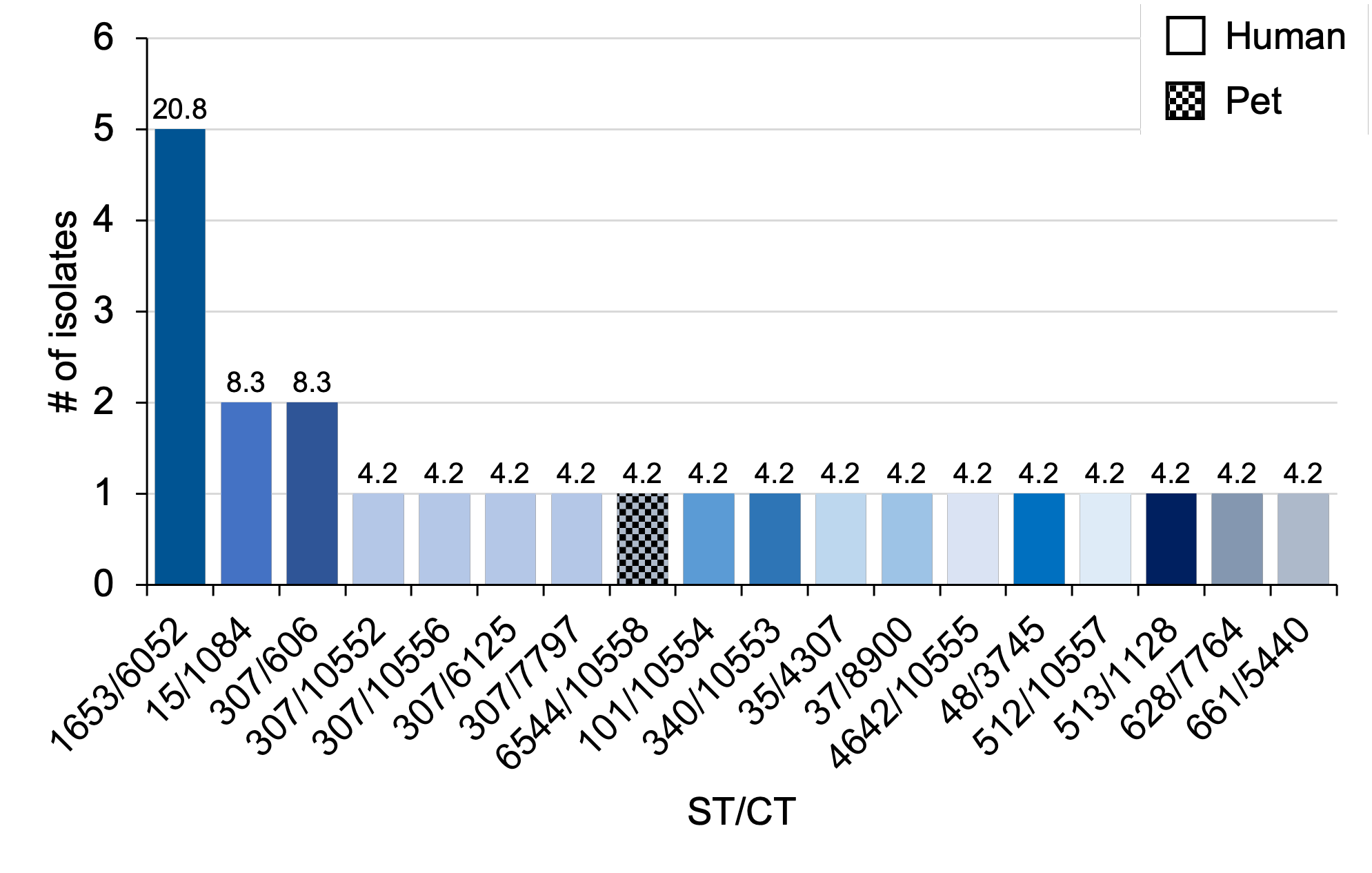

Supplement: Supplementary file 5 — Supplementary Material 5 [file 13756_2024_1457_MOESM5_ESM.tiff]

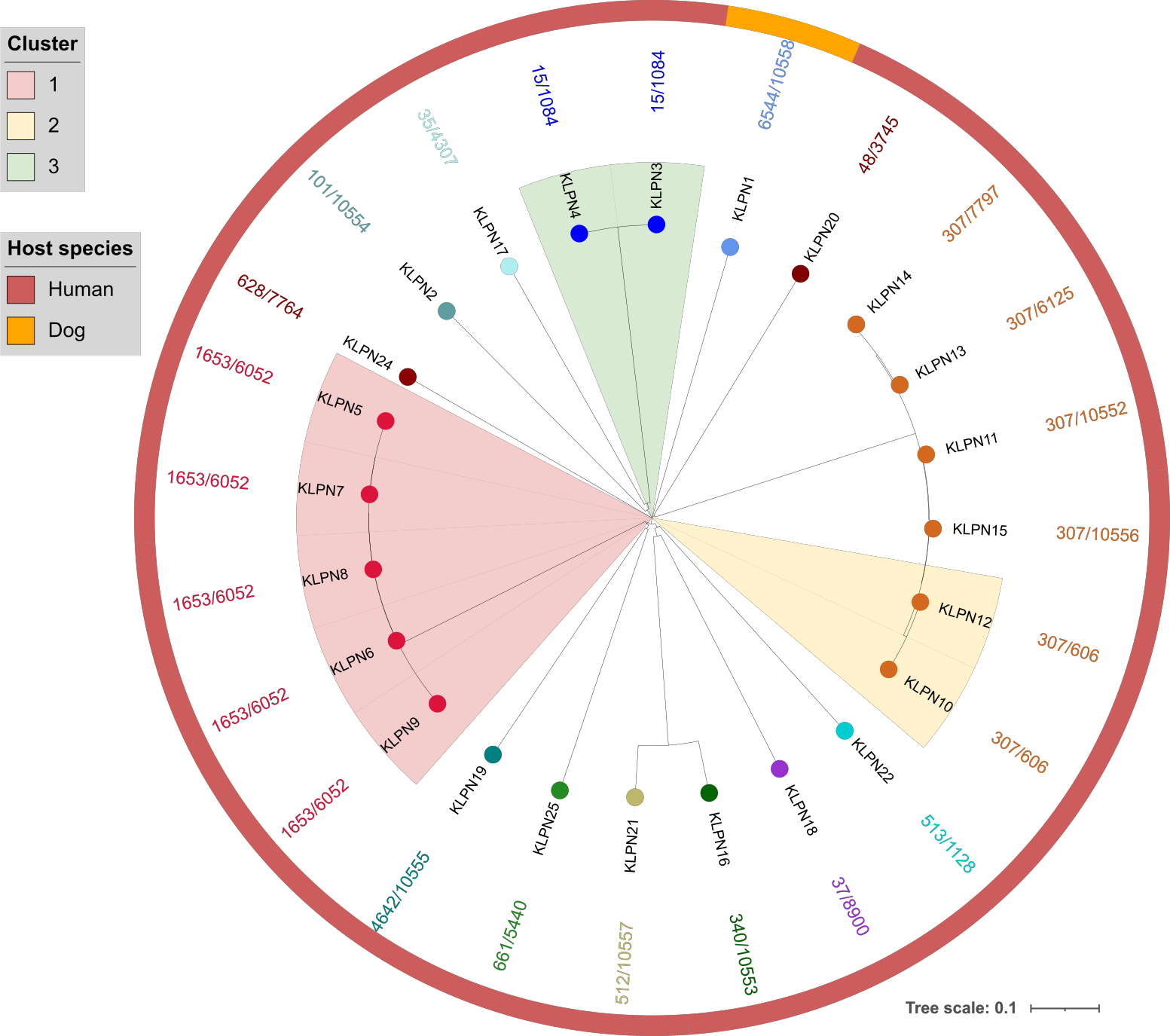

Supplement: Supplementary file 7 — Supplementary Material 7 [file 13756_2024_1457_MOESM7_ESM.tiff]

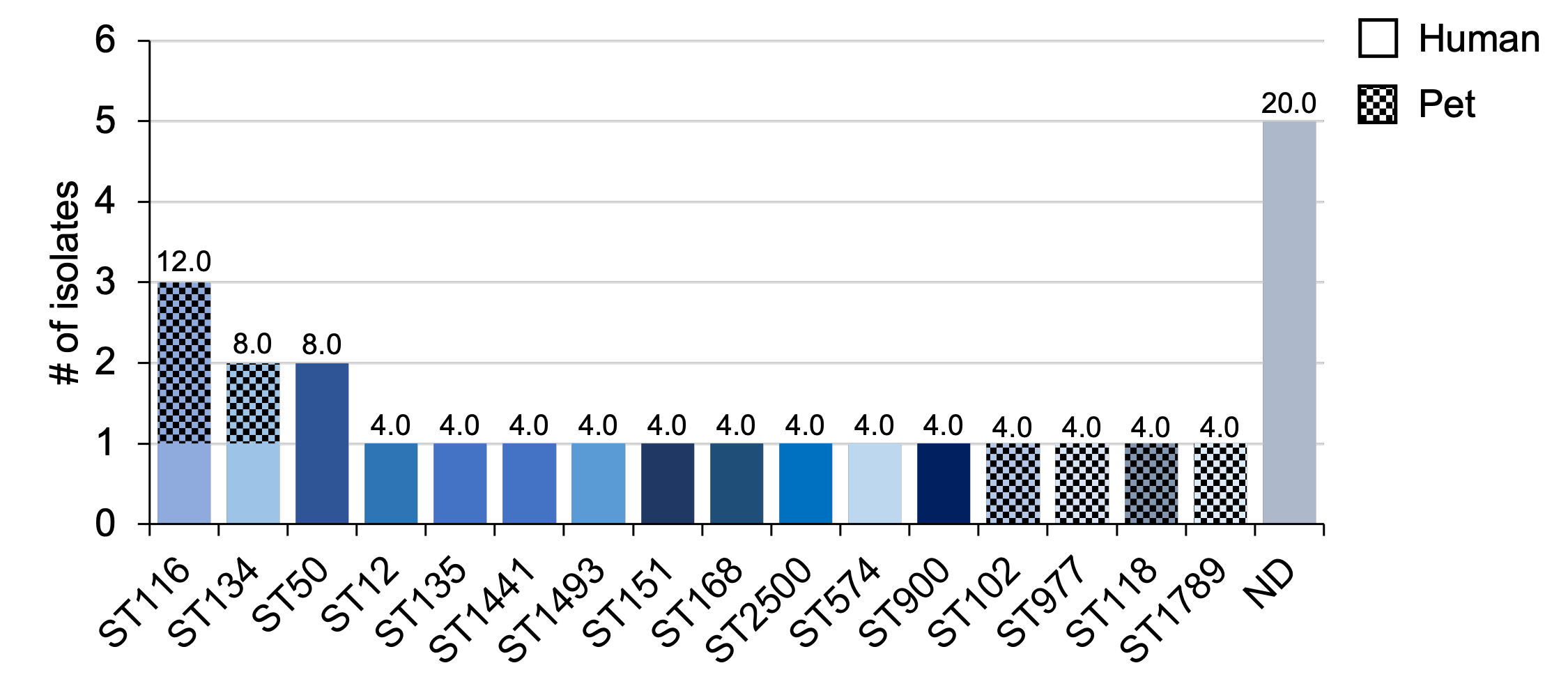

Supplement: Supplementary file 9 — Supplementary Material 9 [file 13756_2024_1457_MOESM9_ESM.tiff]

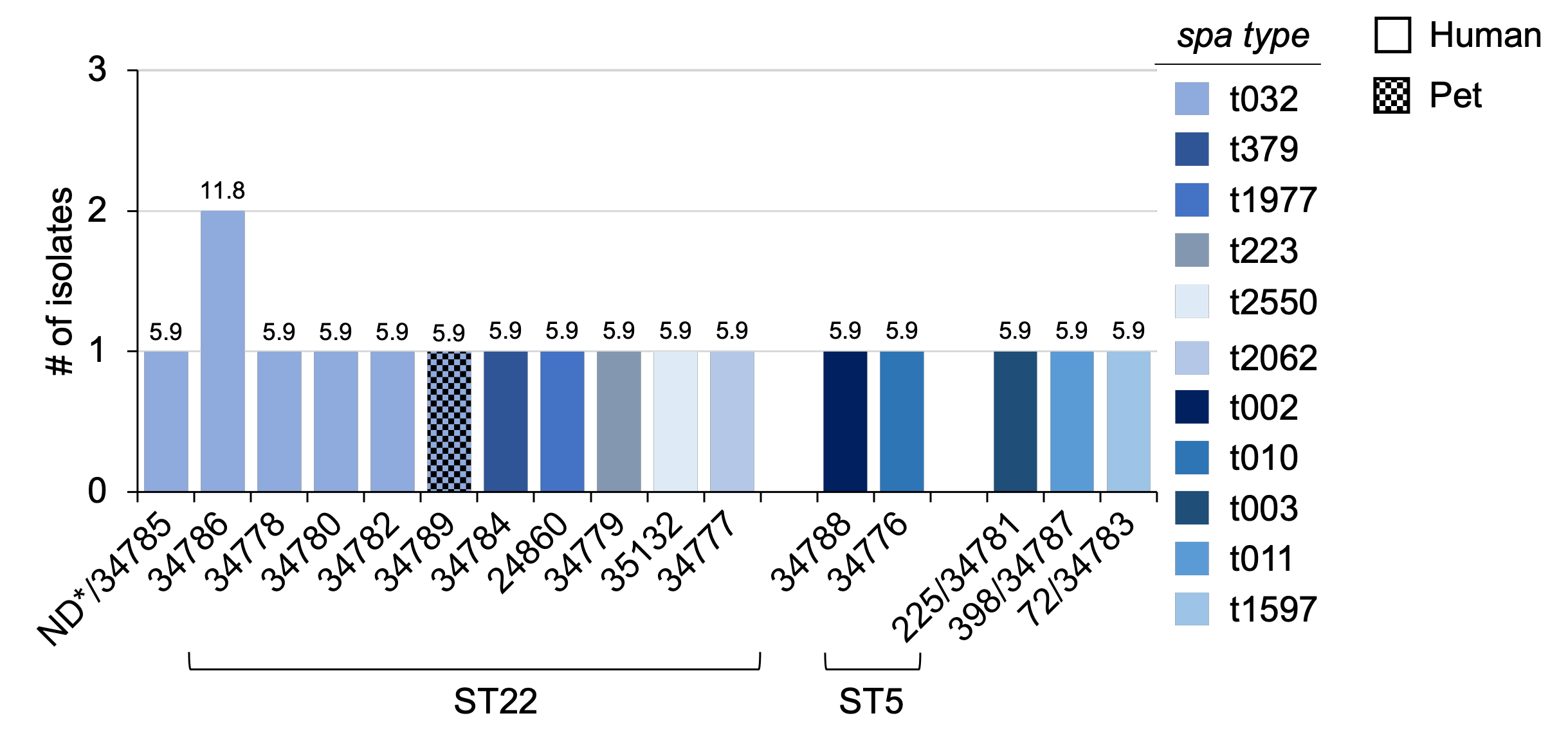

Supplement: Supplementary file 11 — Supplementary Material 11 [file 13756_2024_1457_MOESM11_ESM.tiff]

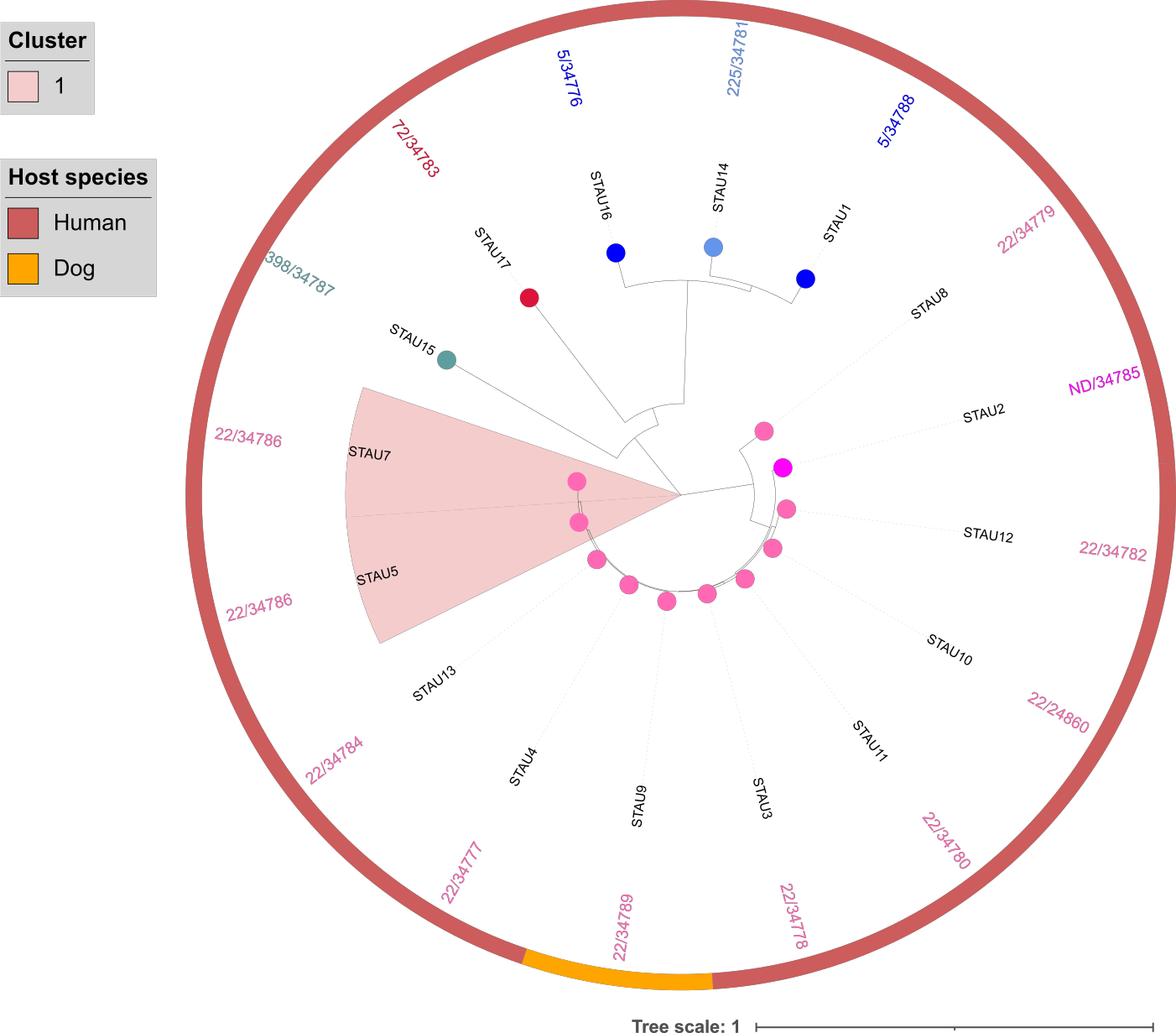

Supplement: Supplementary file 13 — Supplementary Material 13 [file 13756_2024_1457_MOESM13_ESM.tiff]
