# Supplementary material for: The genetic relationship between human and pet isolates: a core genome multilocus sequence analysis of multidrug-resistant bacteria
Source: Antimicrob Resist Infect Control. 2024 Sep 20;13:107. doi: 10.1186/s13756-024-01457-7 (PMC11416027; doi:10.1186/s13756-024-01457-7)
Supplement: Supplementary file 6 — Supplementary Material 6 [file 13756_2024_1457_MOESM6_ESM.docx]

# Additional File 5

S5: Absolute occurrence of sequence types (STs) and complex types (CTs) among all MDR K. pneumoniae isolates according to the cgMLST analysis. The number above the bars indicates the percentage of the respective CT among all isolates. The patterned bar coloring indicates pet isolates.
