# Supplementary material for: The genetic relationship between human and pet isolates: a core genome multilocus sequence analysis of multidrug-resistant bacteria
Source: Antimicrob Resist Infect Control. 2024 Sep 20;13:107. doi: 10.1186/s13756-024-01457-7 (PMC11416027; doi:10.1186/s13756-024-01457-7)
Supplement: Supplementary file 10 — Supplementary Material 10 [file 13756_2024_1457_MOESM10_ESM.docx]

# Additional file 7

S7: Absolute occurrence of sequence types (STs) among all MDR E. cloacae complex isolates according to MLST analysis. The number above the bars indicates the percentage of the respective ST among all isolates. The patterned bar coloring indicates pet isolates. ND = ST could not be determined.
