# Supplementary material for: The genetic relationship between human and pet isolates: a core genome multilocus sequence analysis of multidrug-resistant bacteria
Source: Antimicrob Resist Infect Control. 2024 Sep 20;13:107. doi: 10.1186/s13756-024-01457-7 (PMC11416027; doi:10.1186/s13756-024-01457-7)
Supplement: Supplementary file 12 — Supplementary Material 12 [file 13756_2024_1457_MOESM12_ESM.docx]

# Additional file 8

S8: Absolute frequency of sequence types (STs) and complex types (CTs) among all MRSA isolates according to the cgMLST analysis. The isolates are grouped according to their STs on the x-axis. The individual bars correspond to the CTs. The number above the bars indicates the percentage of the respective CT among all isolates. The coloring of the bars corresponds to the spa type. The patterned bar coloration indicates pet isolates. ND = ST could not be determined but likely corresponds to ST22.
