# Supplementary material for: The genetic relationship between human and pet isolates: a core genome multilocus sequence analysis of multidrug-resistant bacteria
Source: Antimicrob Resist Infect Control. 2024 Sep 20;13:107. doi: 10.1186/s13756-024-01457-7 (PMC11416027; doi:10.1186/s13756-024-01457-7)
Supplement: Supplementary file 14 — Supplementary Material 14 [file 13756_2024_1457_MOESM14_ESM.docx]

# Additional file 9

S9: Neighbor joining tree of the core MRSA genomes from 16 humans and one dog. The phylogenetic tree and cluster analysis were created using SeqSphere+ software and annotated in iTOL v6.8.1. The tree was rooted in the center. Combinations of sequence types (STs) and complex types (CTs) are indicated by colored circles at the leaf nodes. The colored range indicates the cluster. The outer ring represents the host population (human, dog). Cluster Threshold: ≤ 24 allelic differences.
